# Effects of Aluminum Hydroxide and Layered Double Hydroxide on Asphalt Fire Resistance

**DOI:** 10.3390/ma11101939

**Published:** 2018-10-11

**Authors:** Menglin Li, Ling Pang, Meizhu Chen, Jun Xie, Quantao Liu

**Affiliations:** Wuhan University of Technology, State Key Laboratory of Silicate Materials for Architectures, Luoshi Road 122, Wuhan 430070, China; limenglin@whut.edu.cn (M.L.); chenmzh@whut.edu.cn (M.C.); xiejun3970@whut.edu.cn (J.X.); liuqt@whut.edu.cn (Q.L.)

**Keywords:** asphalt combustion, flame retardant, aluminum hydroxide, layered double hydroxide

## Abstract

When a fire occurs in a tunnel, the instantaneous high temperature and smoke cause great danger to people. Therefore, the asphalt pavement material in the tunnel must have sufficient fire resistance. In this study, the effects of aluminum hydroxide and layered double hydroxide on the fire resistance of styrene-butadiene-styrene (SBS) polymer-modified asphalt was investigated. The fire resistance of the asphalt was evaluated by using a limiting oxygen index (LOI). The impact of aluminum hydroxide (ATH), layered double hydroxide (LDHs), and mixed flame retardant (MFR) on LOI was studied. The synergistic fire resistance mechanism of ATH and LDHs in asphalt binder was analyzed by using an integrated thermal analyzer‒mass spectrometry combined system (TG-DSC-MS) and Fourier transform infrared spectrometer (FTIR). The experimental results indicated that the main active temperature range of these flame retardants was 221–483 °C. The main components of smoke were methane, hydroxyl, water, carbon monoxide, aldehyde, carbon dioxide, etc. The addition of flame retardants could inhibit the production of methane, carbon monoxide, and aldehyde. Moreover, due to the good synergistic effects of ATH and LDHs, 20 wt % MFR had the best fire resistance.

## 1. Introduction

Asphalt pavement has been widely used in the construction of highway tunnels due to its excellent road performance and driving comfort [1,2]. Bitumen, as a petroleum product, is mainly composed of hydrocarbons, with an initial decomposition temperature of 200 °C [3]. This indicates that asphalt is flammable. Once a tunnel is ignited, asphalt in the pavement aggravates the spread of the fire. This could cause serious damage to a tunnel road. Therefore, it is important to improve the fire resistance of asphalt.

Adding flame retardants is a popular method of improving the fire resistance of asphalt. Based on previous research, the combustion process of asphalt can be divided into five stages: heating, decomposition, ignition, combustion, and propagation [4]. The addition of flame retardants destroys one or more of these stages [5]. Therefore, many studies have been done to improve the fire resistance of asphalt by adding flame retardants. The existing fire-resistant asphalts are made by adding various organic or inorganic flame retardants. Organic flame retardants have comparable efficiency. Nonetheless, halogen-based flame retardants release toxic smoke. This increases the concentration of poisonous and corrosive gases in the fire, which can easily lead to asphyxiation. Moreover, the amount of smoke released by fire-resistant asphalt mixtures prepared using halogen flame retardants is large, which could seriously affect the health of construction personnel [5,6,7]. For these reasons, inorganic flame retardants have received more attention [8]. As one kind of inorganic flame retardant, hydroxides have the merits of non-toxicity and environmental friendliness [9,10,11,12]. The initial decomposition temperature of both the light components in asphalt and aluminum hydroxide (ATH) is 200 °C [13,14,15]. Therefore, ATH can be used in asphalt to enhance its fire resistance.

LDHs are a family of hydrotalcite compounds that contain intercalated anions, metal cations, and water molecules. LDHs can also be used as a flame retardant in polymers, not only because of its unique chemical composition, similar to aluminum/magnesium hydroxide, but also because of its flake-like morphological structure, which can act as a barrier to heat and fuel transfer. The use of intercalated LDHs to enhance the fire resistance of polymers has been reported. The results showed that the LDHs had the effects of absorbing heat, releasing water, and forming a protective oxide layer that could prevent further degradation [16,17]. When Breu et al. [18,19] used modified LDHs as a flame retardant in polymers, the results showed that the fire resistance of polymers was improved greatly, and the total heat release value of the sample decreased a lot. Zhang et al. [20] combined PWA-LDH with an intumescent flame retardant (IFR) and the results showed that these two flame retardants had a good synergistic effect in promoting char formation and enhancing fire resistance. Xiao et al. [21,22,23] showed that the metal oxides produced by LDH had good inhibiting effects on the emissions of organic volatiles. However, the combination of ATH and LDHs was seldom employed to improve the fire resistance of asphalt.

In this study, a limiting oxygen index (LOI) test was conducted to evaluate the fire resistance of asphalt and flame-retardant-modified asphalt (FRMA). The combined TG-DSC-MS and FTIR techniques were employed to dynamically detect the amount of heat and smoke from asphalt and FRMA in the combustion process. The decomposition regularities of asphalt and FRMA were analyzed. Then the effects of flame retardants on the heat release and smoke suppression in the asphalt combustion were discussed. Finally, synergistic effects between ATH and LDHs were discovered during the combustion process.

## 2. Experimental 

### 2.1. Materials

Styrene-butadiene-styrene (SBS) polymer-modified asphalt was obtained from Jiaotouzhiyuan New Material Industry in Hubei Province, China. Its properties include: SBS dosage of 4.3%, penetration of 5.8 mm at 25 °C, ductility of 57 cm at 5 °C, softening point of 70 °C, and viscosity of 0.95 Pa·s at 135 °C.

The flame retardant, namely ATH, was produced by KeXin Industry of Chemical Limited Company in Wuxi, Jiangsu Province, China. The nontoxic ATH flame retardant was a white powder with an average particle size of 1.5–2.0 μm, density of 2.4 g/cm^3^, and purity of 99.0%.

The flame retardant, namely Mg-Al-LDHs, was produced by TaiKeLaiEr Chemical Industry in Beijing, China. The nontoxic Mg-Al LDHs flame retardant was also a white powder, with an average particle size of 2–20 μm, density of 0.33 g/cm^3^, purity of 99.5%, and ratio of Mg to Al in LDHs of 2:1.

### 2.2. The Preparation of Flame-Retardant-Modified Asphalt

Flame-retardant powders (at a mass percentage of 20%) were added to SBS-modified asphalt at 165 ± 5 °C. First, the blends were stirred with a high-speed shear mixer (ESR-500, ELE, Shanghai, China) at 3000 rpm for 1 h to ensure the uniform distribution of flame retardants. Then, the FRMA was further stirred at a lower speed of 500 rpm for 15 min to reduce the amount of air bubbles. Finally, the FRMA was poured into a clean vessel with continuous hand stirring until it cooled to room temperature. SBS-modified asphalt was processed under the same conditions to ensure comparability with FRMA.

## 3. Methods

An LOI test is used to evaluate the relative combustion of plastics and other polymer materials so as to determine how easily the material can burn when exposed to fire in the air [6]. In order to prepare the specimens for LOI tests, a glass fiber surfacing mat with a density of 120 g/m^2^ was cut into the samples with 120 mm length and 120 mm width; then we put a metal frame of 151 mm length and 151 mm width and 3 mm height on the middle of the glass fiber surfacing mat. After that, uniformly heated asphalt was carefully poured into the metal frame until the whole metal frame was filled up with asphalt. When cooled to room temperature, the asphalt stained with a glass fiber surfacing mat was cut into specimens of 115 mm length and 6.5 mm width for the LOI test. The fire resistance of asphalt was assessed by the limiting oxygen index according to ASTMD-2863-77. The LOI was defined according to Equation (1):

(1) $$\mathrm{LOI}=C_{f}+\mathrm{kd},$$

where LOI is the limiting oxygen index; C*_f_* is the last oxygen concentration; k is a coefficient associated with oxygen concentration; and d is the change in oxygen concentration.

The integrated thermal analyzer‒mass spectrometry combined system (TG-DSC-MS, ATA449F3) used in this study was produced by NETZSCH Group, Bayern, Germany. This technique was used to study the quality changes (TG), heat changes (DSC), and gas composition (MS) caused by chemical reactions and physical changes of samples. About 2.0 mg of the sample was used in each case and the respective peaks were recorded. The sample was heated from room temperature to 700 °C at a heating rate of 10 °C/min under air flowing at 10 mL/min.

The Fourier transform infrared spectrometer (FTIR, Nicolet 6700/Nicolet 6700) used in this study was produced by Thermo Fisher Scientific, Waltham, MA, USA. TG-FTIR experiments were performed using a thermal analyzer system coupled with a Fourier transform infrared spectrometer. The test was conducted under air flowing at 120 mL/min and a heating rate of 10 °C/min. About 10.0 mg of the sample were heated in the TG equipment from room temperature to 700 °C. The mass loss of the sample was recorded. At the same time, the gases released during combustion entered the gas cell for FTIR analysis, and the changes in the products with the temperature were monitored by FTIR spectrometry. The spectrum wavenumber ranged from 4 cm^−1^ to 4000 cm^−1^.

The spectrum intensity was calculated according to Equation (2) or Equation (3):

(2) $$\mathrm{Aborbance}\;\left(\%\right)=\left(1-\frac{I}{I_{0}}\right)\times 100\%$$

(3) $$\mathrm{Transmittance}\;\left(\%\right)=\frac{I}{I_{0}}\times 100\%,$$

where *I*_0_ is the incident light intensity and *I* is the transmitted intensity.

## 4. Results and Discussion

### 4.1. Fire Resistance of Asphalt

The LOI values of SBS-modified asphalt and its composites are shown in Table 1. When the LOI value of the material was greater than 26%, it could be self-extinguished in air [6]. From Table 1, it can be observed that the LOI value of SBS-modified asphalt is just 19.3%, which means that SBS-modified asphalt has good flammability, since the concentration of oxygen in air is about 21% by volume. However, the LOI value of SBS-modified asphalt is obviously improved by the addition of flame retardants. When the content of ATH was 20%, the LOI value was 25%. To meet the requirement of self-extinguishing, the content of ATH in ATH-modified asphalt (AMA) was 25%. The LOI value of LDHs-modified asphalt (LMA) containing 5% LDHs was 20.7%, and the LOI value of 20% LDHs was just 22.5%, which indicated that LDHs had limited effects on the fire resistance of SBS-modified asphalt. The LOI value of mixed flame-retardant-modified asphalt (MMA) with 10% ATH and 7.5% LDHs was 26.0%, which met the requirement of self-extinguishment. In other words, MMA with 10% ATH, 7.5% LDHs, and AMA with 25% ATH had the same effects. It can be concluded that LDHs had good synergistic effects with ATH.

### 4.2. TG Analysis

**Figure 1:**
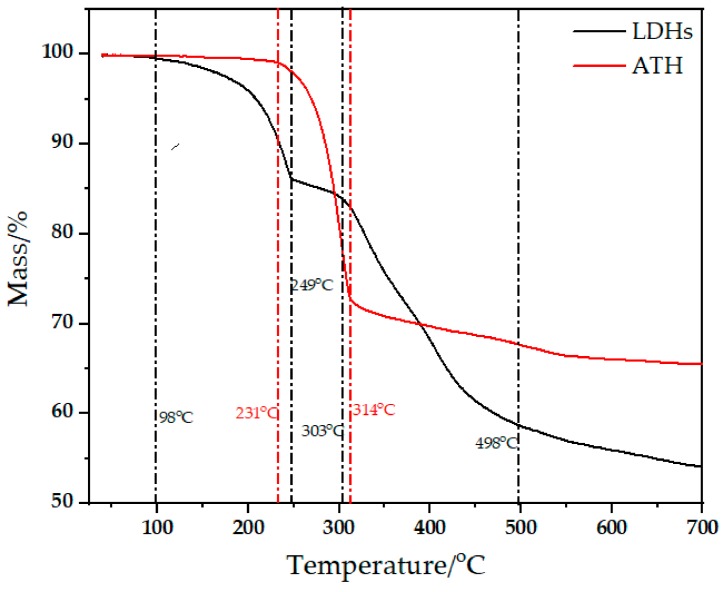
TG curves of LDHs and ATH.

The TG curves of LDHs and ATH are shown in Figure 1. From Figure 1, the main decomposition temperature of ATH ranges from 231 °C to 314 °C. However, LDHs have three decomposition stages: 98~249 °C, 249~303 °C, and 303~498 °C. The stage with the most mass loss has the range 303~498 °C.

**Figure 2:**
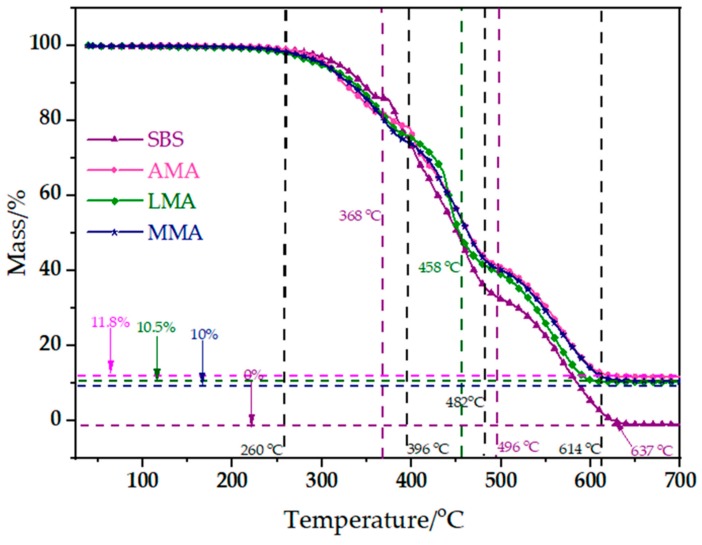
TG curves of SBS-modified asphalt, AMA, LMA, and MMA during pyrolysis.

The TG curves of ATH, LDHs, SBS-modified asphalt, AMA, LMA, and MMA are shown in Figure 2. The whole combustion process can be divided into several stages according to the peaks in the DTG curves.

Due to the complex composition of asphalt, it is hard to study the specific chemical composition. However, the composition can be approximately divided into four components—saturates, aromatics, resins, and asphaltenes—and each component has its own thermal properties.

From Figure 2, the combustion process of SBS-modified asphalt was divided into three stages with temperature ranges from 260 °C to 368 °C, 368 °C to 496 °C, and 496 °C to 637 °C. Stage 1 was from 260 °C to 368 °C. Most of the mass loss in this stage was due to the decomposition of the lightweight components, for example, saturates, aromatics, etc. [9]. In the meantime, due to the complexity of the lightweight components, a mass of volatiles was generated during combustion [24]. Under the combined action of thermal radiation and oxygen, the combustion of these flammable volatiles further promoted the decomposition of bitumen, and a carbon layer begun to form. Stage 2 was from 368 °C to 496 °C. This stage had the most mass loss of asphalt binder, with the most mass loss occurring at 423 °C. The three stages were due to the decomposition of aromatics and resins, and the chemical reactions were more complex [25,26]. In addition, a carbon layer was formed. Finally, Stage 3 was the temperature range from 496 °C to 637 °C. In this stage, the vast majority of bitumen had been decomposed. The most mass loss occurred at 570 °C. The main reason was the decomposition of asphaltenes [25,26]. 

From Figure 2, the decomposition of AMA could also be divided into three stages as the temperature ranged from 260 °C to 396 °C, 396 °C to 482 °C, and 482 °C to 614 °C, just as with SBS-modified asphalt. The general trend of the AMA curve was similar to SBS-modified asphalt, but there were some differences. Compared with SBS-modified asphalt, the temperature range of AMA was much larger. In addition, the first stage could be divided into two smaller stages by the remaining mass of asphalt. The first small stage was at the temperature from 260 °C to 388 °C, and the other one was at 388 °C to 396 °C. The most mass loss in the first small stage occurred at 316 °C, which was close to ATH. In this small stage, the mass loss of AMA was greater than that of SBS-modified asphalt. The main reason for this was the decomposition of ATH in AMA. Moreover, the decomposition of ATH was an endothermic reaction, and the decomposition product Al_2_O_3_ could also promote the formation of the carbon layer, which had an adverse effect on the decomposition and combustion of the lightweight components of asphalt in this stage. In the second stage, the undecomposed lightweight components were fully decomposed, which released a large amount of flammable volatiles. The burning of these volatiles further promoted the decomposition of bitumen, which also promoted the formation of a carbon layer. As a result, the char yield ratio of AMA was increased by 11.8 wt % to generate a tight carbon layer. 

Figure 2 shows that decomposition of LMA also could be divided into three stages with temperature ranges from 260 °C to 396 °C, 396 °C to 458 °C, and 458 °C to 614 °C. In the first stage, the mass loss of LMA was greater than that of SBS-modified asphalt, but after 396 °C, the mass loss of LMA was smaller than that of SBS-modified asphalt, which was very similar to the trend of AMA. However, there were some notable differences between LMA and AMA. Firstly, the curve of AMA and SBS-modified asphalt only intersected at one point, while the curve of LMA and SBS asphalt intersected twice. Secondly, the second stage of LAM ranged from 396 °C to 458 °C, while that of AMA ranged from 396 °C to 482 °C. Thirdly, the first stage of LAM only had one process, but there were two small processes in the first stage of AMA. Finally, the char yield ratio of LMA was 10 wt %, while that of AMA was 11.8 wt %. Since the LDHs used for modification were first treated by sodium stearate, the first two differences might be caused by the presence of sodium stearate in the reaction. Moreover, the third difference indicated that LDHs had a greater temperature impact in the first stage than ATH, which meant that LDHs had a better inhibitory effect on the decomposition of lightweight components of asphalt. 

From Figure 2, the decomposition of MMA could also be divided into three stages with temperature ranges from 260 °C to 396 °C, 396 °C to 482 °C, and 482 °C to 614 °C. Compared with LMA, the curve tendency of MMA was closer to that of AMA. There was only one process in the first stage of MMA, the curve of MMA and SBS-modified asphalt only intersected at one point, and the char yield ratio of MMA was 10.5%. As the MMA modifier contained ATH and LDHs, there were some interactions between ATH and LDHs during the whole combustion process, which eventually led to these results.

In conclusion, the combustion process of SBS-modified asphalt was mainly divided into three stages, and the flame retardants mainly affect the first two stages. The main functions of the flame retardants include: decomposing and absorbing heat, inhibiting the decomposition of light components in asphalt, and promoting the formation of a tight carbon layer.

### 4.3. DSC Analysis 

**Figure 3:**
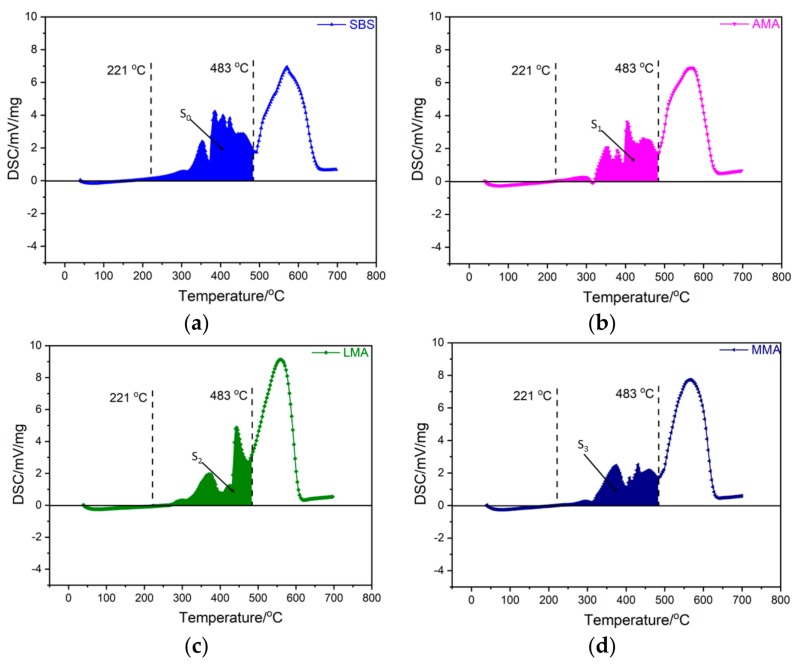
Results of DSC test of SBS-modified asphalt (**a**), AMA (**b**), LMA (**c**), and MMA (**d**).

DSC technology could be used for thermal analysis during combustion. The DSC test results of these asphalts are represented in Figure 3a‒d. According to the TG results, the main active temperature range of flame retardants was 200–500 °C. Therefore, in the DSC analysis, heat released within the temperature range 221 °C to 483 °C was the main analysis target. The area of the curve within this temperature range was used to represent heat. The larger the area, the more heat was released in this temperature range. S_0_ was the heat released by SBS-modified asphalt; S_1_ was the heat released by AMA; S_2_ was the heat released by LMA; and S_3_ was the heat released by MMA.

The values of S_0_ to S_3_ are shown in Table 2.

As seen from Table 2, S_0_ > S_1_ > S_2_ > S_3_, which meant that in th temperature range, SBS-modified asphalt releases the most heat, followed by AMA, then LMA, and finally MMA. This implied that the addition of flame retardants significantly reduced the heat released in this temperature range.

In order to better distinguish the effects of these three flame retardants, the flame retardant index (*HI*) is used in this study. The *HI* was defined as in Equation (5):

(4) $${\Delta S}_{n}=\left|S_{n}-S_{0}\right|\;$$

(5) $$HI_{n}=\frac{{\Delta S}_{n}}{S_{0}},$$

where *HI_n_* is the flame retardant index of *n*th flame retardant; ΔS*_n_* is the heat difference between nth flame-retardant-modified asphalt and SBS-modified asphalt; and S_0_ is the heat released by SBS-modified asphalt.

Table 3 shows the values of *HI_n_* and the heat differences.

Table 3 shows that *HI*_3_ > *HI*_2_ > *HI*_1_, indicating that mixed flame retardants reduced heat the most when compared with the other two single flame retardants in this temperature range. Moreover, the mixed flame retardant was prepared by these two single flame retardants with a mass ratio of 1:1. So, ATH and LDHs had a good synergy effect.

### 4.4. FTIR Analysis

**Figure 4:**
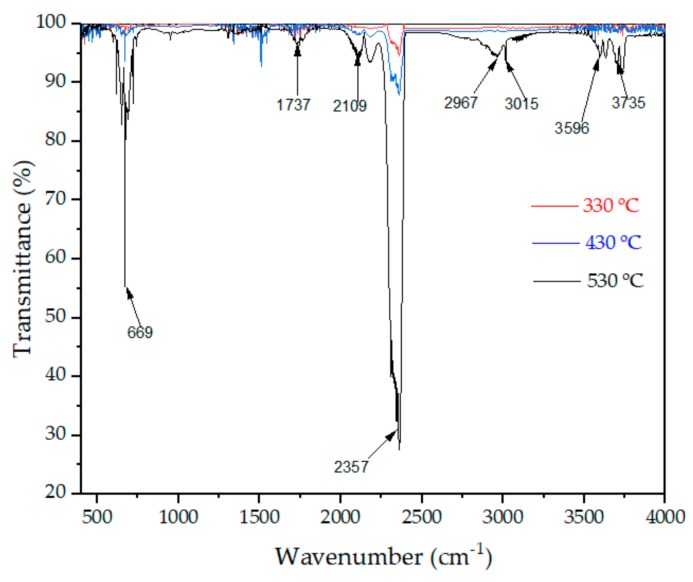
FTIR spectra of volatile products during the asphalt binder combustion process.

Figure 4 shows the infrared results of volatiles from SBS-modified asphalt. The components of the volatiles were characterized by the peak position in the infrared spectrogram. The peaks at 2357 cm^−1^ and 669 cm^−1^ were the absorption peaks of carbon dioxide. The peak at 2357 cm^−1^ was the asymmetrical stretching vibration absorption peak, and the peak at 669 cm^−1^ was the bending vibration absorption peak. The peak at 1737 cm^−1^ was the characteristic peak of THE carbonyl group, which meant aldehyde compounds might have been produced. The peaks at 2109 cm^−1^ were the absorption peaks of carbon monoxide. The peak at 2967 cm^−1^ was the asymmetric stretching vibration absorption of methyl. The peak at 2930 cm^−1^ was the asymmetric stretching vibration absorption of methylene, and the peak at 2860 cm^−1^ was the symmetric stretching vibration of methyl. The peak at 3015 cm^−1^ was the absorption peak of methane. All of these peaks showed that there were alkanes contained in flammable gases, and the gases were the main components for further combustion of asphalt. The peaks within the range from 3500 cm^−1^ to 3775 cm^−1^ were caused by the stretching vibration of the O‒H bond, which indicated that the smoke contained water vapor.

In conclusion, methane and carbon dioxide are the main components of greenhouse gases in smoke. Both carbon monoxide and aldehyde are toxic and are the main sources of toxicity in smoke. Hence, the products of the asphalt combustion process not only cause great harm to the environment, but can also seriously affect human health, especially in the case of a tunnel fire.

### 4.5. MS Analysis

**Figure 5:**
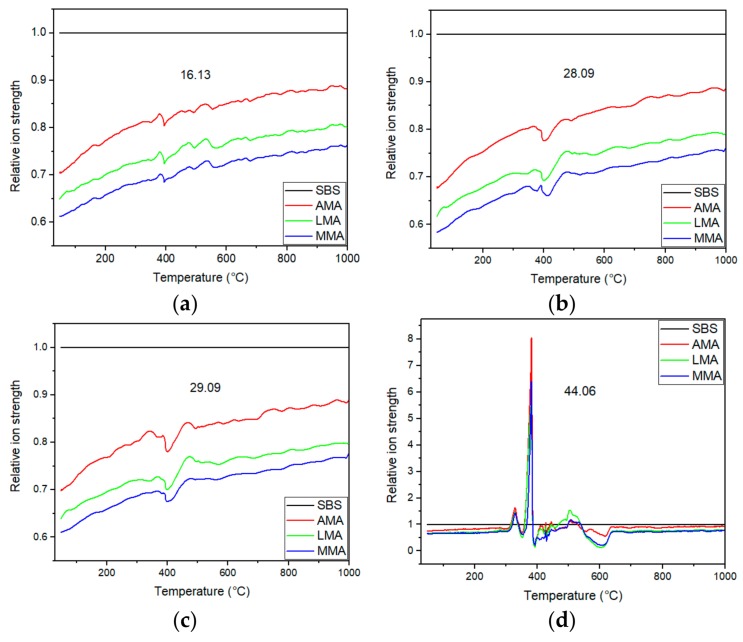
Relative ionic strength of four major components: 16.13 (**a**), 28.09 (**b**), 29.09 (**c**), and 44.06 (**d**).

MS technology was used to study the effect of flame retardants on the composition of bitumen smoke. Figure 4 shows that the main components of bituminous gas were carbon dioxide, aldehyde, carbon monoxide, alkanes, methane, and water vapor. Therefore, in the MS test, the ion strength of 16.13, 28.09, 29.09, and 44.06 relative molecular mass was mainly detected. These relative molecular masses represented methane, hydroxyl, water, carbon monoxide, aldehyde, and carbon dioxide, respectively. In order to better study the antismoking effect of flame retardants, the results of SBS-modified asphalt were standardized based on the research results. The relative ion strength of each component is shown in Figure 5.

It could be concluded from Figure 4 and Figure 5a–c that the addition of these three flame retardants could effectively inhibit the production of methane, carbon monoxide, and aldehyde. The order of inhibition ability was: MIX > LDHs > ATH. Compared to ATH, LDHs has a good layered structure, which has a good promoting effect on the formation of carbon layer. Therefore, LDHs have a better antismoking effect than ATH. It could also be seen from the DSC analysis that ATH and LDHs had a good synergy effect. This synergy effect was also confirmed in the MS results. Reducing the content of methane in asphalt flue gas is of great importance for reducing greenhouse gas emission. The reduction of carbon monoxide and aldehyde can reduce the toxicity of asphalt flue gas, which is of great importance during evacuation and subsequent rescue. However, compared to the other three smoke components, these three flame retardants had little effect on carbon dioxide (Figure 5d). On the whole, flame retardants not only hindered the burning of asphalt, but also inhibited the production of smoke.

## 5. Conclusions

The following conclusion can be obtained from the analysis of the experimental results:

(1) The combustion of SBS-modified asphalt could be roughly divided into three stages. The main active temperature range of these flame retardants was 221–483 °C, which covers the first two stages. The addition of flame retardants could significantly reduce the heat released within this temperature range, among which mixed flame retardants were the most effective, reducing the heat release by 34.89%.
(2) The main components in smoke emitted were: carbon dioxide, aldehyde, carbon monoxide, alkanes, methane, and water vapor. Aldehyde and carbon monoxide were the main sources of smoke toxicity, and carbon dioxide and methane are the main greenhouse gases. The addition of flame retardants significantly reduced the content of aldehydes, carbon monoxide, and methane, which indicated that the flame retardant could also inhibit smoke emission.
(3) The main role of the flame retardant in SBS-modified asphalt was to decompose and absorb heat, and to inhibit the decomposition of lightweight components in the initial stage of combustion. Secondly, the decomposition products of flame retardants were inorganic metallic oxides, which could promote the formation of a carbon layer in the course of asphalt combustion.
(4) Due to the good synergistic effect of ATH and LDHs, the mixed flame retardant has the best flame-retardant and smoke-suppression effects.

## Figures and Tables

**Table 1:** LOI data of SBS-modified asphalt and its composites.

Name of Material	LOI (%)
SBS	19.3
SBS + 5% ATH	20.5
SBS + 10% ATH	21.7
SBS + 15% ATH	23.2
SBS + 20% ATH	25.0
SBS + 25% ATH	26.6
SBS + 30% ATH	28.4
SBS + 5% LHDs	20.7
SBS + 10% LDHs	21.3
SBS + 15% LDHs	21.6
SBS + 20% LDHs	22.5
SBS + 10% ATH + 2.5% LDHs	23.4
SBS + 10% ATH + 5% LDHs	24.6
SBS + 10% ATH + 7.5% LDHs	26.0
SBS + 10% ATH + 10% LDHs	27.3

**Table 2:** Values of DSC test results for 221–483 °C.

S_0_	S_1_	S_2_	S_3_
455.35	327.63	318.19	296.47

**Table 3:** Values of flame retardant indexes.

*HI*_1_	*HI*_2_	*HI*_3_
28.05%	30.12%	34.89%
